# Panel Doctors and Panel Chemists

**Published:** 1914-10-31

**Authors:** 


### THE PROFESSION OF MEDICINE.

### Panel Doctors and Panel Chemists.

### THE PRACTICAL REMEDY FOR OVER-PRESCRIBING.

In consequence of the fact that the sums allotted for the payment of drugs and appliances have not been sufficient to pay the chemists' accounts in full in quite a considerable number of areas the Insurance Commissioners for England have been making inquiries with a view to ascertaining the causes of the deficiencies. As a result of their investigations the Commissioners have come to the conclusion that a considerable portion of the charges upon the drug funds " could have been avoided without loss of efficiency, and even with positive advantage to insured patients, by properly directed economy in prescribing." This very definite statement, implying as it does a charge of careless or extravagant prescribing on the part of sections of panel doctors, is a serious matter, and in a memorandum addressed to Insurance Committees the Commissioners point out that " they cannot contemplate with equanimity the continuance of a system under which, through the absence of effective control, an unnecessary burden is cast upon Insurance funds, and, through them, upon the funds of approved societies." Reading between the lines of this memorandum it clearly seems to be the intention of the Commissioners to take steps to stop the drain upon the funds due to this cause. If this cannot be done by means of the machinery provided by the existing regulations it will be necessary to strengthen the machinery.

#### Where Present Remedies Fail.

Under the present provisions an Insurance practitioner, against whom a charge of excessive prescribing is proved, can be surcharged with the amount of the excess, but it is no easy matter to put these provisions into force. Since the panel chemists are financially interested in seeing that the drug fund in any area is properly used, and any deficit being a certain loss to them, it is only right that the regulations should authorise them to set the machinery in motion; but to charge a doctor or a collection of doctors with extravagant prescribing is not a task which the chemists relish, and hence they are apt to be slow in taking the initiative.

But it has, however, been made quite clear to them that however distasteful the duty may be they can expect no sympathy from the Commissioners unless the duty is punctually performed, and in consequence of this intimation the Pharmaceutical Committees, especially in districts where there is a shortage in the drug funds, are scrutinising the prescriptions very carefully with a view to reporting any cases of extravagance to the Panel Committees for investigation. But it is important that Insurance practitioners should note that it is open to the Panel Committees to conduct an investigation at any time without waiting to be prompted by the Pharmaceutical Committees.

In view of the possibility of the Commissioners making drastic alterations in the regulations providing for the allocation of the medical benefit funds it is urgently necessary for Panel Committees to do everything that is expedient and possible to prevent wastage.

#### A Possible Drastic Measure.

At present the amount available for the payment for drugs and appliances is at the rate of two shillings per insured person, but apparently there is nothing to prevent the Commissioners from enacting provisions whereby no definite sum would be set aside for the payment of the chemists, but which would give the chemists the first call on the fund. Under such an arrangement the drug accounts would be paid in full and the balance would go to the panel fund. No doubt the Commissioners would be reluctant to make such a change in the regulations, but if there were no other means of stopping extravagant prescribing they might be compelled in the interests of all concerned to take this step.

Therefore, in the long run it will be as much to the interests of panel doctors as of panel chemists if there is properly directed economy in prescribing. That extravagant practices exist has undoubtedly been proved. Investigations made by the Commissioners disclose differences in the cost of the prescribing, both as between individual practitioners on the same panel and as between different panel areas, of such magnitude that they cannot be satisfactorily explained either by a difference in the health average of the areas or the individual practitioner's lists, or by legitimate variations in the standard of prescribing observed by individual practitioners.

This evidence, we must admit, strongly suggests that a minority of practitioners are prescribing on a scale or in a manner which is extravagant, judged by the standard of their colleagues. This memorandum should be read in conjunction with another just issued by the Scottish Commissioners which gives some examples of methods of prescribing which may reasonably be called extravagant; for instance, one practitioner ordered 2 lb. of compound lobelia powder at one time; another gave five prescription orders in a month, the five bottles as prescribed containing more than two quarts of pure alcohol, while the cost of the five orders was respectively, £1 14s. 6d.; 17s. 4d.; 15s. 9d.; 14s. 3d.; and 14s. 3d.; or, in all, £4 16s. 1d. Another practitioner frequently ordered at one time 4 lb. of petroleum emulsion, and the same doctor frequently prescribed 6 oz. of glyco heroin at one time. It is needless to multiply instances. It should be borne in mind that over-prescribing is only practised by the minority, and it therefore behoves the majority on the lines suggested above to ensure that the whole scheme is not jeopardised.

